# A Web-Based Dementia Education Program and its Application to an Australian Web-Based Dementia Care Competency and Training Network: Integrative Systematic Review

**DOI:** 10.2196/16808

**Published:** 2020-01-22

**Authors:** Anne Moehead, Kathryn DeSouza, Karen Walsh, Sabrina W Pit

**Affiliations:** 1 Northern New South Wales Local Health District New South Wales Ministry of Health Lismore Australia; 2 Western Sydney University Sydney Australia; 3 University Centre for Rural Health University of Sydney Sydney Australia

**Keywords:** education, workforce, online learning, Web-based learning, distance education, dementia, nursing, facilitated learning, competency, training, network, capability, skills, person-centered

## Abstract

**Background:**

Dementia education that meets quality and safety standards is paramount to ensure a highly skilled dementia care workforce. Web-based education provides a flexible and cost-effective medium. To be successful, Web-based education must contain features that promote learning and support knowledge translation into practice. The Dementia Care Competency and Training Network (DCC&TN) has developed an innovative Web-based program that promotes improvement of the attitudes, knowledge, skills, behavior, and practice of clinicians, regardless of their work setting, in order to improve the quality of life for people living with dementia.

**Objective:**

This review aims to (1) determine the key features that are associated with an effective and functional Web-based education program—an effective and functional Web-based program is defined as one that measures results, is accessible, is user friendly, and translates into clinical practice—and (2) determine how these features correlate with the DCC&TN.

**Methods:**

Six electronic databases—Medline, Embase, Cumulative Index to Nursing and Allied Health Literature (CINAHL), AusHealth, Nursing@Ovid, and Google Scholar—were searched for articles published between 2009 and 2018 using the following keywords: Education, Distance, Continuing, Learning, Online, Web-Based, Internet, Dementia, Program Evaluation, Validation Studies, Outcome and Process Assessment Healthcare, Nursing, Assisted Instruction, and Facilitated. The Critical Appraisal Skills Programme (CASP) and Kirkpatrick’s model for the evaluation of training were used to ensure quality and rigor of the analysis.

**Results:**

A total of 46 studies met the inclusion criteria. In total, 14 key features were associated with an effective Web-based learning environment, which enabled the environment to be as follows: self-directed, individualized, interactive, multimodal, flexible, accessible, consistent, cost-effective, measurable with respect to participant satisfaction, equitable, facilitated, nurturing of critical thinking and reflection, supportive of creating a learning community, and translated into practice. These features were further categorized into five subgroups: applicability, attractiveness, functionality, learner interaction, and implementation into practice. Literature frequently cites Kirkpatrick’s four-level model of evaluation and application in the review of education and training; however, few studies appeared to integrate all four levels of Kirkpatrick’s model. Features were then correlated against the DCC&TN, with an encouraging connection found between these features and their inclusion within the content and structure of the DCC&TN.

**Conclusions:**

A total of 14 key features were identified that support an effective and functional Web-based learning environment. Few studies incorporated Kirkpatrick’s salient elements of the model—reaction, learning, behavior, and results—in their evaluation and clinical application. It could, therefore, be considered prudent to include Kirkpatrick’s levels of training evaluation within studies of dementia training. There were few studies that evaluated Web-based dementia education programs, with even fewer reporting evidence that Web-based training could increase staff confidence, knowledge, skills, and attitudes toward people with dementia and be sustainable over time. The DCC&TN appeared to contain the majority of key features and is one of the few programs inclusive of hospital, community, and residential care settings. The 14 key features can potentially enhance and complement future development of online training programs for health sciences education and beyond. The DCC&TN model could potentially be used as a template for future developers and evaluators of Web-based dementia training.

## Introduction

### Background

The global dementia epidemic demands a skilled and knowledgeable workforce ready to meet its related challenges [1]. For clinicians, there is an urgent need for access to education that is user friendly, affordable, and accessible with available peer support, supervision, and access to dementia champions, especially for those working in rural and remote regions. Web-based education has changed the face of learning and provides flexible, accessible, and cost-effective platforms for the delivery of education to a wide audience, regardless of their setting or location [2].

In 2015, an estimated 46.8 million people were living with dementia globally. The number of people living with dementia is expected to reach 74.7 million by 2030 and 131.5 million by 2050. In high-income countries, this number will grow by 116% between 2015 and 2050 [3]. In 2018, the estimated cost of dementia to Australia was over Aus $15 billion [4]. People with dementia occupy up to one-quarter of Australian acute-care hospital beds [1,5] and make up 52% of all residents in residential aged-care facilities [4].

The lack of professional knowledge around treatment and care options can result in delayed or hindered access to ongoing care, treatment, and support for people living with dementia [1,5]. Web-based education provides a platform for health professionals to access flexible education to improve awareness, knowledge, and skills in delivering dementia care. The high enrollment rate—almost 10,000 people from 65 countries—in the Understanding Dementia Massive Open Online Course (MOOC) [6] highlights the interest and need for quality dementia education. However, the 38% completion rate of the MOOC [6] was relatively low, which reflects the need for a more effective and responsive learning environment.

In 2007, a dementia Web-based program—the Dementia Care Competency and Training Network (DCC&TN)—was developed based on recommendations from a report commissioned by New South Wales (NSW) Health by Wylie et al [7,8]. This program aims to advance the knowledge, skills, and practice of clinicians and is facilitated by dementia champions. Our definition of a dementia champion is a “clinician who has excellent knowledge and skills in the care of the person with dementia and has a commitment to provide information and support to those undertaking the online courses.” The DCC&TN delivers a Web-based learning platform that is interactive, multimodal, and facilitated. Additionally, the program includes dementia care competencies developed by Traynor et al [9,10]. These competencies are available to enhance the learner’s knowledge, skills, and attitudes in the delivery of person-centered care and are freely available. Prior to the development of these competencies, Traynor and coworkers reported that no dementia competency framework existed that was applicable across care settings or levels of practice [10].

Since the inception of the DCC&TN, it has delivered high-quality dementia education and resources to over 10,000 clinicians across NSW, Australia, with an average completion rate of 78%. The program is a key training resource for NSW dementia clinical nurse consultants with a focus on a person-centered approach to delivering dementia care. The syllabus content of the DCC&TN aligns with the Australian Commission on Safety and Quality in Health Care Standards [5], with the goal of embedding these standards into clinical practice to improve the quality of life and outcomes for people living with dementia.

The literature suggests that Web-based education is a flexible [6,11-14] and cost-effective [6,15-18] medium; however, to be effective and functional it must contain features that promote learning and support knowledge translation into practice [19-21]. To our knowledge, there is limited research that has evaluated Web-based dementia education programs. An effective and functional Web-based program is defined as one that measures results, is accessible, is user friendly, and translates to clinical practice.

The DCC&TN provides a multifaceted education platform that assists clinicians in meeting the challenges of caring for the person living with dementia and in meeting professional obligations for lifelong learning. The training network is an online website comprising a content management system and a learning management system integrated into a single user experience that provides continuous membership, allowing ongoing access to resources, tools, clinical experts, and interactive forums. The library includes resources referenced within five online courses.

### Objectives

This review aims to (1) determine the key features that are associated with an effective and functional Web-based education program, which is defined as one that measures results, is accessible, is user friendly, and translates into clinical practice, and (2) determine how these features correlate with the DCC&TN.

## Methods

### Overview

Integrative review methodology [22] was chosen for several reasons. First, it allows for the inclusion of both experimental and nonexperimental research, so as to improve our understanding of a phenomenon. Second, integrative reviews can also combine empirical literature together with theoretical frameworks [22]. Lastly, integrative reviews address a diverse range of purposes, including defining concepts, reviewing theories or evidence, and analyzing methodological issues. The wide-ranging sampling frame, together with the diversity of purposes of integrative reviews, can assist in understanding complex concepts and theories, such as Web-based dementia care education. Hence, this review included both qualitative and quantitative studies as well as literature that looked at theories and empirical studies [22].

### Study Selection

Six electronic databases—Medline, Embase, Cumulative Index to Nursing and Allied Health Literature (CINAHL), AusHealth, Nursing@Ovid, and Google Scholar—were searched for studies published between 2009 and 2018. Keywords used included the following: Education, Distance, Continuing, Learning, Online, Web-Based, Internet, Dementia, Program Evaluation, Validation Studies, Outcome and Process Assessment Healthcare, Nursing, Assisted Instruction, and Facilitated (see Multimedia Appendix 1). The Critical Appraisal Skills Programme (CASP) [23] and Kirkpatrick’s salient elements of the model for the evaluation of training were used to ensure quality and rigor of the analysis [18]. Search strategies were carried out using Medical Subject Headings (MeSH), relevant terms citations, and abbreviations (see Multimedia Appendix 1). Citation tracking and reference list inspections were undertaken in the search for relevant papers. Three clinical experts (KDS, AM, and KW) in dementia education conducted three stages of study selection: (1) an initial screening of titles, (2) a review of titles and abstracts, and (3) a review of the full text to identify suitable articles for inclusion. Consensus was used in the case of discrepancies.

### Eligibility Criteria

#### Inclusion Criteria

Studies had to adhere to the following inclusion criteria in order to be included in the review:

1. Population: health personnel across different care settings or levels of practice.
2. Concept: educational interventions that included (1) Web-based learning, online learning, Internet-based education, or computer‐assisted instruction, and (2) interactive facilitated education or tutor-supported education. Studies also had to measure learner satisfaction, knowledge, skills, or behavior.
3. Context: articles from countries with similar health care provision to Australia were included.
4. Types of studies: quantitative and qualitative research papers were included. Additionally, comparison studies, literature reviews, case studies, cohort studies, systematic reviews, and randomized controlled trials were included. Studies that contained experiential and correlational designs, quasi designs, flexible learning, and Web-based learning were also included.

#### Exclusion Criteria

Exclusion criteria were as follows: studies that excluded information technology (IT), studies published prior to 2009, studies that were unobtainable, non-English literature, studies with unrelated relevance to the review objectives, studies with nonhealth-related context, protocol descriptions, and studies with limited reporting on translation of learning or outcomes.

### Quality Appraisal, Abstraction, and Synthesis

The CASP system of appraisal [23] was adopted to evaluate the final studies for rigor, methods, credibility, and relevance. CASP is a well-utilized tool to enhance the utility of evidence-based research by health professionals. Each article was critiqued for design, methods, and study detail including aims, ethical considerations, sample population and size, interventions, and outcome measures (see Textbox 1).

### Data Extraction

A standardized data extraction process to perform data extraction was used. Any discrepancies were resolved by consensus. Data extracted from each eligible study was entered into a standardized form and included the following:

1. General information: author, year of publication, and location.
2. Study characteristics: aim, study design, and ethics.
3. Sample population and size.
4. Comparative interventions.
5. Outcome measures and instruments.
6. Main findings.

A rating criteria framework was developed and agreed upon by the authors (see Textbox 1) to compare all studies so that recurring key features could be identified and subsequently applied during the review. These criteria also included questions on possible correlation of the studies with the components and characteristics of the DCC&TN.

**Rating criteria applied across the 46 articles included in the review.**

- Correlation to the Dementia Care Competency and Training Network (DCC&TN): strong, medium, or weak
- Findings identified with evidence and in context
- Key concepts and aims
- Contribution to a wider understanding of online learning
- Lessons learned from literature and application to the DCC&TN
- Methods, omissions analysis, and validity
- Research conclusions to the objectives
- Learner satisfaction

### Association of Key Features With the Dementia Care Competency and Training Network

Following the review, 14 key features were compared with the DCC&TN. Correlation with the features found in the review and the content and delivery of the DCC&TN was considered to be *strong* if eight or more features matched the DCC&TN, *medium* if between four and seven features matched the DCC&TN, and *weak* if three or fewer of the features matched the DCC&TN.

### Ethics Approval and Consent to Participate

Ethics approval from a human research ethics committee was not required, as this was a systematic review of published literature.

## Results

### Search Outcome

**Figure 1:**
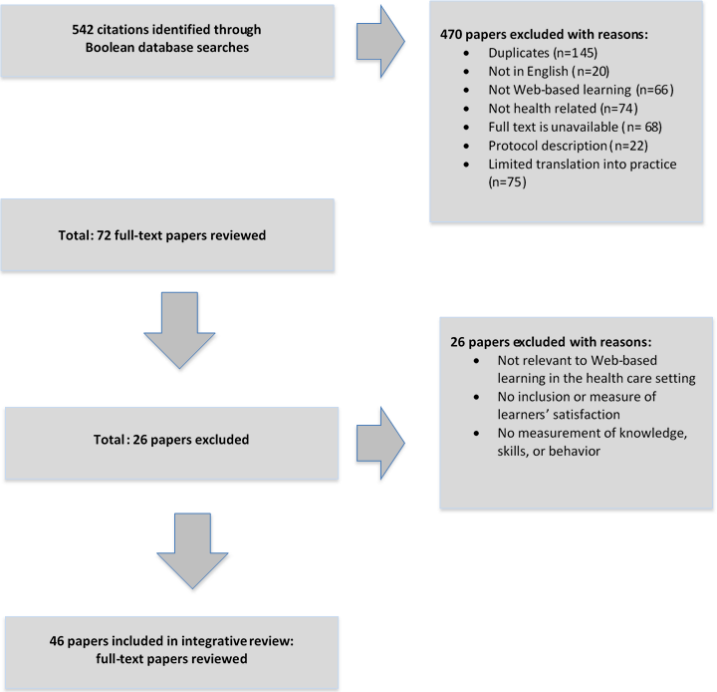
Flow diagram of the systematic review search.

There were three phases of the search process. Initially, the systematic search produced 542 citations; 470 were excluded for the following reasons: duplication; non-English; full text unavailable; relevance to objectives; context not health related; not inclusive of, or not delivered via, the Internet or not Web based or online; protocol descriptions; and limited reporting on translation of learning or outcomes (see Figure 1). Secondly, 72 full-text papers were subsequently reviewed; of these, an additional 26 papers were excluded. The third phase included the review of the remaining 46 articles, which were matched to the rating criteria in Textbox 1. The review of articles included the following study designs: 6 randomized controlled trials, 30 cross-sectional studies, and 10 literature reviews.

### Setting and Participants

Multimedia Appendix 2 displays a summary of the 46 included papers [6-56]. Most studies were conducted in the United States (13/46, 28%), followed by the United Kingdom (10/46, 22%) and Australia (8/46, 17%), with the remaining 33% (15/46) from a variety of countries. Web-based training programs were delivered to health professionals.

### Data Synthesis of Key Features

The qualitative synthesis of the 46 articles was structured on what features were linked to an effective and functional Web-based learning experience and how this correlates with the DCC&TN. During data analysis, two initial observations were made. First, one seminal systematic review published in 2009 by Booth et al [14] identified five broad themes: peer communication, flexibility, support, knowledge validation, and course presentation and design. This was further supported by 11 subthemes, which provided a valuable framework for ongoing course development, as suggested by Booth et al and the literature.

Second, 6 articles referred to Kirkpatrick’s salient elements of the four-level model, which are applied to determine “return on investment” and to “show [that] the business value and worth of training” was used to evaluate training and education provision [12,18,37,56]:

1. Level 1: Examines the learner’s reaction to and satisfaction with the program.
2. Level 2: Assesses the extent of learning and includes knowledge, skills, confidence, and attitudes.
3. Level 3: Explores the extent to which completion of the training leads to staff behavior or practice change.
4. Level 4: Assesses the results or outcomes of training, for example, in terms of quality of patient care.

Surr and Gates [19,38], Ellis et al [12], and Scerri et al [39] refer to Kirkpatrick’s salient elements of the model of reaction, learning, behavior, results, and outcomes. Surr and Gates also reported that it is a weakness not to consider Kirkpatrick’s framework within dementia education [38].

Upon further analysis of the 46 articles, 14 key features were identified and applied to what were considered effective and functional features for Web-based learning programs (see Table 1 and Multimedia Appendix 2). Some key features were used more frequently in studies, such as *self-paced* or *self-directed* *learning* (n=29) and the program being interactive (n=28). Key features that were used less frequently included *cost-effectiveness* (n=10), *provides equitable engagement* (n=13), and *individualized, based on learner’s profile and background* (n=15).

In total, 5 studies reported that training can increase staff confidence, knowledge, and attitudes toward those with dementia [20,21,33,39,40]. Gagnon et al [33] reported that the use of self-directed educational modules improved nurses’ knowledge and skills in relation to evidence-based practice. Du et al’s [21] systematic review identified learners as having a high satisfaction rate in regard to Web-based education as well as having improvement in knowledge and skills performance and enhanced self-efficacy in accomplishing nursing skills. Very few studies reported completion rates or whether learnings were translated into clinical practice. The themes that emerged from the review could be incorporated into future design elements of dementia-related Web-based learning programs. Some examples include the following:

1. Delivery of an entire learning experience that goes beyond the module and syllabus design [17,19,24].
2. Development of learning communities [13,21,31,41,45].
3. Connecting learning to improved clinical practice [15,17,26,28,38,54].
4. Learning that leads to the best possible provision of care for people living with dementia [17,21-49].

A number of papers highlight barriers faced by online learners, including lack of time, competing interests, reliability of IT, organizational support during work hours, computer access, confidence with the computer, and ability to work at their own pace [19,38,44]. It is important that these factors are considered in the development of Web-based learning. For example, issues such as lack of time, competing interests, and organizational support may potentially be addressed by content design, which includes short, sharp training measures in the way Web-based education is delivered.

It was frequently reported that learners who are engaged in Web-based studies require a level of commitment and willingness, the ability to develop self-direction, and a capacity for flexibility [14,19-37,42,43]. The authors’ evaluation of the learners’ feedback regarding the DCC&TN emphasized similar challenges.

### Application of Key Features to the Dementia Care Competency and Training Network Program

Following the literature review, the 14 key features were cross-referenced with the syllabus content, the interactive elements of the learning platform, and the delivery environment of the DCC&TN. Table 1 shows that all features were found to correlate with the program; some features correlated strongly while others had only a weak correlation. Of the 46 studies reviewed, 57% (26/46) had a strong correlation, 28% (13/46) had a medium correlation, and 15% (7/46) had a weak correlation to the DCC&TN. Overall, the features identified in the literature, which are required for a functional and effective Web-based learning environment, are embedded in the content and structure within the learning management system of the DCC&TN.

**Table 1:** Thematic analysis of the literature identifying key features for effective and functional online learning.

Theme number	Key feature	Number of articles^a^ that included the key feature, n	Application of key feature to the Dementia Care Competency and Training Network (DCC&TN)
1	Self-directed and self-paced	29	Learners choose when and where to engage in the courses at a time that suits their personal commitments
2	Individualized and based on learner’s profile and background	15	Learners can choose courses based on their interests, competencies, and experiences
3	Interactive	28	Learner interaction occurs via real-time live chats and forums
4	Multimodal	22	Interactive and multimodal Web-based lessons are delivered, including video, interaction via the Web, and literature
5	Flexible	23	Interaction and learning is available at a time that suits the learner
6	Accessible	23	The program develops a dementia community of learners as they are enrolled in individual groups, regardless of location; the program has a user-friendly format
7	Consistency of information, repetition, and reinforcement	21	Questionnaires, feedback, surveys of satisfaction, and case discussions are embedded throughout courses and are reinforced by current literature and clinical champions
8	Cost-effective and good value for investment, both for the learner and the system	10	All courses are free and available to anyone who applies, regardless of professional background or geographical location; established education program is for use by educators and facilitators
9	Measures using questionnaires, feedback, and surveys of satisfaction	28	Various feedback mechanisms are utilized, including SurveyMonkey, learner satisfaction comments, and pre-post questionnaires
10	Provides equitable engagement	13	All courses are free and available to anyone who applies, regardless of professional background or geographical location
11	Facilitated, access to instructor, or mentored	25	Dementia facilitators support learners by providing weekly updates and encouragement, undertaking grading, and responding to individual emails for those learners who have not completed their learning milestones
12	Nurtures critical thinking and reflection	26	Reflective practice occurs via forum posts and discussion
13	Establishment of a learning community	22	Learners interact in real-time chats and forum posts, sharing their professional and personal experiences, providing case discussion, and sharing of clinical procedures and policies among their course group, thereby consolidating a learning community
14	Ability for translation into practice	17	Learners are encouraged to undertake activities or projects that demonstrate translation of learning into practice within the workplace

^a^See Multimedia Appendix 2 for individual references.

## Discussion

### Principal Findings

This literature review identified key features that could contribute to effective and functional Web-based learning programs. The literature frequently cited Kirkpatrick’s model of evaluation and application in the review of education and training, identifying four significant elements: reaction, learning, behavior, and results. These four elements align with the expanded 14 features identified in this literature review. Additionally, the review suggests that the structure and content of the DCC&TN nurtures critical thinking within a learning community, via support and facilitation by dementia champions, and ultimately encourages translation of learning into effective person-centered practice. This has been further substantiated by the evaluation of the participant’s response to feedback questionnaires during the learning experience. The DCC&TN achieves completion rates of 78% each year compared to, for example, 38% in the MOOC dementia education training program [6]. The DCC&TN has also aligned its syllabus content and resources with the Australian National Safety and Quality Health Service Standards of clinical care.

Surr and Gates [38] conducted a systematic review on effective dementia education and training for the health and social care workforce. They concluded that none of the reviews examined each of the salient elements of Kirkpatrick’s four levels of evaluation. They also found that none of the reviews combined the elements to understand the full context of key features that lead to an efficacious dementia training program in the hospital setting. This was determined to be a weakness in the current dementia literature [19]. In total, 8 studies in this review highlighted the importance of including the evaluation of staff confidence, knowledge, and attitudes toward those living with dementia [19,21-49]. Additionally, the majority of studies did not report on completion rates or whether clinical practices could be sustained over time [20,54]. The authors suggest that these are important features for translating learning into practice and should be incorporated into future evaluations. This review indicates that there is still limited evidence on the effectiveness of training in changing staff confidence, knowledge, and attitudes [37,39].

This analysis also identified a shortfall, in that much of the literature does not consider the importance of the following identified themes: training design, content or a delivery mode that is self-directed and individualized [14,38], interactivity [6,14], multimodality [6,14], flexibility [17,36,54], accessibility [17,36,54], consistency [6], and cost-effectiveness [6,17]. The findings indicate that greater depth and breadth of knowledge and education is needed to have an impact on clinicians’ feelings of caring efficacy, positive attitudes, and satisfaction toward people with dementia [13,19,39,40,47,49,54].

Many of the 14 features were found across the literature in varying degrees, with Booth et al [14] identifying five similar themes and Kirkpatrick’s four salient themes, further validating the 14 key features linked to an effective Web-based education environment, as follows: self-directed, individualized, interactive, multimodal, flexible, accessible, consistent, cost-effective, measurable with respect to participant satisfaction, equitable, facilitated, nurturing of critical thinking, supportive of creating a learning community, and translated into practice. The DCC&TN is able to demonstrate correlation in varying degrees to each of these features. The DCC&TN has established a *real-world* application of what is fundamental to enable translation of dementia education to the clinical coalface.

### Barriers

The literature reports that Web-based learners are challenged by the following: lack of time, busy workplace, reliability of IT, limited organizational support during work hours, computer access, confidence with computers, and the ability to work at their own pace. Learners require a strong commitment to external studies, requiring a level of discipline, a willingness to develop self-direction, and a capacity for resilience [14,19-37,42,43]. It is the authors’ opinion that a work-life balance needs to be addressed to encourage online learning among health professionals, their peers, and their managers.

### Strengths and Limitations

Some of the excluded articles could have contributed further in emphasizing limitations for effective dementia Web-based training. The selection of papers reviewed were diverse and provided a good overview for comparison. Caution should be used when interpreting the results in order to consider outcomes that may have been reported elsewhere outside of this review.

### Recommendations and Future Research

First, the DCC&TN can potentially provide an effective professional development platform and simultaneously advocate that improved outcomes for people living with dementia can be achieved with effective, functional, Web-based training programs.  The ideal outcome for any Web-based dementia program is to improve the quality of life and well-being of people with dementia through the delivery of person-centered care by a skilled and knowledgeable workforce. Therefore, future developers seeking to design and develop new and innovative Web-based learning programs for dementia clinicians could be well-inspired by the exemplary DCC&TN structure and delivery mode. Second, clinicians require better access to free learning opportunities to be informed and competent in knowledge translation [57]. Our DCC&TN is provided for free to clinicians to learn about dementia person-centered care and how to apply learned knowledge to practice. Third, there are many apps that offer functions that have resulted in reducing the burden and improving health outcomes of caregivers [58]. It will be interesting to conduct further research to identify whether the same 14 features are important for training family caregivers in an online environment. Fourth, currently there is an Aged Care Royal Commission in place in Australia. The Australian Government has responded to one of the key recommendations that has come out of the Commission and has announced that it will deliver Aus $10 million for additional dementia training and support for aged-care workers and providers [59]. The results from our study can potentially be used to assist in delivering this key recommendation. Fifth, the International Organization for Standardization aims for harmonization of products and services globally. Recently, a technical committee has been set up in the area of aging societies [60]. The results from this study can potentially be used to inform standardization processes in the area of online dementia training.

### Conclusions

This review identified 14 key features that are linked to deliver a functional and effective online dementia learning environment. The DCC&TN demonstrates correlation in varying degrees to each of the identified key features required for an effective and functional Web-based learning environment in that it delivers a platform that is self-directed, individualized, interactive, multimodal, flexible, accessible, consistent, and cost-effective. It is suggested that critical thinking is nurtured within a learning community supported by dementia facilitators, while encouraging translation of learning into practice.

## Appendix

Multimedia Appendix 1 Example of a search strategy to identify papers for this review.

Multimedia Appendix 2 Summary of all included studies.

## Abbreviations

CASP: Critical Appraisal Skills Programme
CINAHL: Cumulative Index to Nursing and Allied Health Literature
DCC&TN: Dementia Care Competency and Training Network
IT: information technology
MeSH: Medical Subject Headings
MOOC: Massive Open Online Course
NSW: New South Wales
