# Supplementary material for: A Web-Based Dementia Education Program and its Application to an Australian Web-Based Dementia Care Competency and Training Network: Integrative Systematic Review
Source: J Med Internet Res. 2020 Jan 22;22(1):e16808. doi: 10.2196/16808 (PMC7003124; doi:10.2196/16808)
Supplement: Multimedia Appendix 2 [file jmir_v22i1e16808_app2.doc]

| Authors, date and location | Study Design &  Aim/s | Ethical Considerations | Population size | Comparative interventions | Outcome measures / instruments | Main Findings |
| --- | --- | --- | --- | --- | --- | --- |
| Goldberg L, Bell E, King C, O’Mara C, McInerney F, Robinson A, Vickers J.  2015. | Process evaluation | Ethics approval for the study was obtained from the Tasmanian  Social Science Human Research Ethics Committee  (ref: H0013173). | 9,538 registrants  4409 registrants engaged | None noted | - No. of discussion posts made by participants - No.of ‘lerkers’ (those who did not make posts) - No. who participated - No. % who completed | The completion rate and level of engagement of participants across a broad spectrum of education levels suggest that MOOCs can be successfully developed and delivered to students from diverse educational backgrounds.  The high participation rate also highlights the combination of MOOC design as well as the scale of unmet need for quality dementia education |
| Related theme no.  1, 3, 4, 5, 6, 7, 8, 9, 10, 12 | AIM:  To highlight the completion rates and participation rates of engagement |
| Shatto B, Erwin K. 2016. | Literature review | Not included | None noted | Only 4 other articles were located on learning styles on generation X outside of that written by marketing research firms | Literature search.   - Review of mobile technology tablets or smart phones and apps when possible - Encourage collaboration through the use of social media sites - Reinforce concepts with You Tube videos and interactive games - Limit readings to include only necessary information - Include discussions on inclusiveness and tolerance | Generation Z students have unique learning characteristics and preferences.  They are self-directed learners who thrive on technol­ogy.  Traditional lecture-format classes may not engage students and the assigned readings may not be completed as with previous generations.  Today’s teacher must interact more and lecture less.  By creating a dy­namic learning environment, nurse educators will be able to help Gen­eration Z student |
| Related theme no.  1, 2 | AIM:  Looks at unique learning char­acteristics of Generation Z  De­scribe innovative teaching strategies to engage this new breed of student |
| McGowan B, Balmer J, Chapell K.  2014. | Descriptive study | Not included | 102 learners took  178 notes  set 408 reminders  searched through a library of nearly 100 collated and related resources. |  | - Series of videos created - Learners were given access to eLearning environment one month before the symposium - During the month they received weekly notes of encouragement | Learners participating in a flipped learning experience perceive themselves as being more prepared to participate and engage in live or classroom-style learning.  Four weeks of weekly notifications seemed sufficient to drive high levels of participation, although many participants noted that they waited until the final 96 hours to ensure the content was fresh in their minds.  Not all learners will participate in pre-work, with clear and effective communication, a more structured and engaging online learning experience, and sufficient incentives (such as additional continuing education credits), it is possible to surpass 80% participation.  Connecting the flipped video series appears to be a critical success factor. |
| Related theme no.  1, 3, 7, 11 | AIMS:  To maximize learning opportunities in a 1-day conference  To expose nurse  educators to the flipped classroom model as a potential tool  To expand the number of contact hours by combining pre-work with classroom experience. |
| Ballman K, Garritano N, Beery T.  2016. | Technological review | Not included | Development team made up of Nurse researcher instructional designer  IT expert | Standardised patients are used in many NP programs as an adjunct to real patient encounters in clinical settings.  This has been found to be an effective teaching strategy | - The interactive experience allowing students to engage with an SP (standardised patient) is delivered in a step-by-step Web-based format. - Students enter information about their confidence with differential diagnosis, they then view a video with a subject matter expert (SME). - Filming takes place from a vantage point that allows the distance learner to have a first-person experience. - The student is introduced to an SP with a specific complaint the student develops and documents appropriate questions for obtaining a focused history, the students then compared their findings and rationales with those of the SME | Learners at a distance have the same educational needs as the on-campus student.  Providing alternate methods for experiential learning and incorporating SP encounters can improve the quality of education and enhance the competence and confidence of NP students in distance learning programs |
| Related theme no.  3, 4, 5, 7, 10, 11, 12, 14 | AIM:  To determine if there was a difference in students’ ability to identify differential diagnoses. |
| Hardy S, Mushore M, Goddard L. 2016. | Mixed methods | Students reminded of confidentiality | 2 academic institutions  Student nurses in their final year of nursing | Little has been trialed or tested in how to utilise online technology to provide nursing students to gain access to tutors via computer based online tutorials. | - All participants completed an evaluation data sheet using a five-point Likert scale and free text evaluation - Feedback form completed at the end of each online tutorial session. - Students were also invited to a focus group and all tutors were interviewed at the completion of the project | The VIPS project findings highlight: the importance of a clear project vision for innovation uptake; Consequences of working with innovation champions; How technology can be used to maximise student learning across geographical distance through online-facilitated group critical discussion.  VIPS' participants were able to articulate positive outcomes as a result of engaging in a multi-institutional project that capitalised on the richness of nursing clinical practice learning experience for both the students and the academics involved as innovation champions |
| Related theme no.  6, 9, 11, 12, 13 | AIM:  To improve the pace of innovation uptake in encouraging  use of enhanced technology in nurse education,  To ensure others  can also maximise on identified benefits |
| Maloney S, Nicklen P, Rivers G, Foo J, Ooi Y, Reeves S, Walsh K, Ilic D.  2015. | RCT | Nil noted | A total of 497 students were randomized to receive EBM teaching  via either the incumbent face-to-face approach (F2F) or the Blended Learning approach.  147 (29.6%) of the 497 students completed the follow-up assessments on EBM competency and attitudes. EBM  competencies were assessed using the validated Berlin  questionnaire | Research has shown that web-based learning results in improved outcomes when applied to health professional education, with studies focusing on clinical disciplines within medicine reporting an increase in student self-efficacy, knowledge and self-directed learning. | - This study used validated outcome measures of EBM competency to determine that BL is no more effective than F2F at increasing medical students’ knowledge and skills in EBM - For an educator to effectively review their practices and pedagogy, they must consider the learning experience and learning outcomes alongside measures of cost and value | Under the study conditions, a Blended Learning approach was more cost-effective to operate and resulted in improved value for the institution after the third-year iteration, when compared to the traditional face-to-face model.  The wider applicability of the findings is dependent on the type of blended learning utilized, staffing expertise, and educational context |
| Related theme no.  1, 2, 3, 4, 6, 8, 9, 12, 14 | AIM:  To determine the cost-effectiveness of a face-to-face learning and blended learning approach for  Evidence-based medicine training within a medical program. |
| Bourhy H, Troupin C, Faye O, Meslin F, Abela-Ridder B, Sall A, Kraehenbuhle J.  2015. | Evaluation of training | None noted | Onsite workshops over 12 days  24 teachers from 7 countries  106 applicants  32 trainees / participants | The current most popular e-learning systems for resource poor settings are massive online open courses, used by tens of thousands of students around the globe.  This format is not well suited for specific practical training needs. | - Online pre-training assessment - Three activities - Annotated articles - Modules to study - Team exercise - Onsite workshop over 12 days | Customized online training is suitable for disease-control programmes in low-income countries.  Online pre-workshop activities are essential for preparing participants for the workshop.  Mentoring is needed to create a strong network of disease-control experts working in similar settings. |
| Related theme no.  1, 2, 3, 4, 7, 9, 10, 11, 12, 13, 14 | AIM:  To provide practical training on rabies pre­vention in Africa for students and professionals in animal and human public health sectors. |
| Peine A, Kabino K, Spreckelsen C. 2016. | Mixed method trial | The consent of the RWTH Aachen Faculty of Medicine, represented by the leader board of RWTH Aachen University Modellstudiengang and all responsible course directors, was obtained in advance of the study. | 244 initial participants  223 completed the respective module and were included in the study. | A study comparing learning outcomes and student satisfaction in a classroom setting versus an online instructional course does not show significance differences in learning outcomes, but reveals generally high acceptance and student satisfaction levels. | - Students were assigned to one of four study branches representing self-instructed learning forms (e-learning and curriculum-based self-study) and instructed learning forms (lectures and seminars). - All groups participated in their respective learning module with standardised materials and instructions. - Learning effect was measured with pre-test and post-test multiple-choice questionnaires. - Student satisfaction and learning style were examined via self-assessment. | The study shows that students in modern study curricula learn better through modern self-instructed methods than through conventional methods.  These methods should be used more, as they also show good levels of student acceptance and higher scores in personal self-assessment of knowledge. |
| Related theme no.  1, 7, 9, 11, 12 | AIM:  To compare extant traditional teaching methods with new instruction forms in terms of learning effect and student satisfaction |
| Law M, Rapoport M, Seitz D, Davidson M, Madan R, Wiens A.  2015. | Evaluation report | Institutional research ethics board granted ethics approval | 5 study groups with 10 participants each, balanced in years of practice, geography and academic/community settings  50 participants  29 completed the questionnaire | Further research is needed to  identify factors influencing uptake, especially in the context of CPD with a diverse group  of participants with varied expertise, experience, and learning style preferences | - Midway through 2012-2013 5 study groups were consolidated into three as some groups were not as active in online discussions - 22 distinct modules, each lasting 14 days - A retrospective web survey assessed self-efficacy, knowledge in geriatric psychiatry, comfort with online learning, and perceived effectiveness of the instructional methods. - Wilcoxon signed-rank tests and descriptive statistics were calculated. | The OSG was well-received, with greater benefits for self-efficacy with the material and comfort with online learning than for perceived knowledge itself.  Further research is needed to ascertain actual knowledge change in the context of online learning in medical education. |
| Related theme no.  2, 9, 12, 13 | AIM:  An evaluation results of an online study group (OSG) for geriatric psychiatry continuing professional development. |
| Nguyen T.  2015. | Literature review | None noted | A systematic review of various papers that compared the format hybrid and blended learning and purely online learning.  Much of the literature compares blended learning and online learning against traditional face to face learning.  55 references in reference list | Both hybrid or  blended learning and purely online learning are considered to be online learning as much of the literature  compares these two formats against the traditional face-to-face | - There is strong evidence to suggest that online learning is at least as effective as the traditional format, but the evidence is, by no means, conclusive. | There is strong evidence to suggest that online learning is at least as effective as the traditional format, but the evidence is, by no means, conclusive.  Online learning is a story that is still being written, and how it progresses will likely depend on those present. |
| Related theme no.  3, 5, 6, | AIM:  To what extent does the body of work on online learning indicate that online learning is as least as effective in educating students as the traditional format? |
| Canniford L, Fox-Young S.  2013. | Evaluation and descriptive review | This project was intended to enhance the scholarship of teaching and learning within an existing programme of undergraduate nursing study.  Students were advised that their comments would not have any effect on their progress in the course, as they would not be read until after grades were determined | 163 participants were students in their first semester in an Australian undergraduate nursing program.  This under-graduate nursing program involved two consecutive sessions per week in the clinical setting and the online learning modules were designed to complement that clinical exposure | Various approaches to teaching and assessing reflective practice reviewed.  Professional educators are divided about how to best evaluate students learning of reflective practice | - The students evaluated the learning modules and the blog assessment separately using online Likert scale questionnaires. - The module evaluation followed immediately after completion of each module. | The outcomes of this project indicate the need for further research to confirm whether student learning on reflection is enhanced more by timely feedback or by guided online learning modules.  The mediating effects of the online learning process and individual learning styles also need to be further explored. |
| Related theme no.  1, 3, 4, 5, 6, 7, 9, 10, 11, 12, 13, 14 | AIM:  To describe the introduction and evaluation of an innovative, integrated, interactive approach to teaching and assessing competence in reflective practice using an online self-directed learning package |
| Boyd M, Baliko B, Polyakova-Norwood V.  2015. | Evaluation | None noted | Interactive debates to teach EBP skills in a large (200+ students) online undergraduate course. | American Association of College of Nursing review of research expectations for various levels of nursing | - All online courses in the authors’ program are designed with the same format, and the teaching approach emphasizes interactivity and prompt feedback on all learning activities. - At the core of the design are weekly course guides, which provide students with a list of activities and resources necessary for each week, including readings, lectures, quizzes, and surveys. - Students always know what is expected, of them in any given week and have ready access to the tools necessary to complete all tasks. | Students remain highly engaged while practicing critical thinking, teamwork, leadership, delegation, communication skills, and peer evaluation through participation in a series of faculty facilitated online debates. |
| Related theme no.  1, 3, 4, 5, 6, 7, 9, 10, 11, 12, 13, 14 | AIM:  The purpose of this article is to  describe the authors’ experience in successfully implementing  an innovative teaching–learning strategy. |
| Gagnon J, Gagnon M, Buteau R, Azizah G, Jette S, Lampron A, Simonyan D ,Asua J, Reviriego E. 2015. | Evaluation report  International collaboration | Approval for the study was obtained from the Ethics in  Human Experimentation Committee of a university hospital center in Quebec, Canada.  For the Spanish study, ethical approval was not required given that the study was an evaluation of a learning activity.  Written informed consent was obtained from each participant.  All the information related to the project was kept confidential; only members of the research team had access to it. | The target population was composed of nurses with a basic  knowledge of research methodology from a university hospital  center in Quebec, Canada (n 36), and from different areas of the Basque Health Service, Osakidetza (n 47).  Participation in the study was voluntary, and participants  received continuing education credits for completing modules  A prospective pre -post study was conducted with 36 nurses from  Quebec and 47 from the Basque Country.  Assessment in order to explore the main intervention outcomes: knowledge acquisition and self-learning readiness.  Satisfaction was also measured at the end of the course. | Two systematic reviews that consisted of 201 studies that compared the effects of internet-based learning to no intervention and to non-internet interventions | - Before the course, nurses were asked to complete two questionnaires: the knowledge questionnaire and the SDLRSNE. - After the course, they were asked to repeat the same two questionnaires and, in addition, to fill out a final satisfaction questionnaire. - This questionnaire also included open-ended questions allowing participants to comment on different aspects of the modules. | Of the 58 nurses initially recruited in Quebec, only 36 completed the training, despite the incentives proposed  Overall the study found an online self-learning course for teaching critical appraisal skills and evidence-based practice, developed through international collaboration, was implemented and evaluated among practicing nurses in Quebec and the Basque Country.  The results provide support for the use of e-learning to increase nurses’ knowledge of research methodology, as well as a critical approach to decision making based on scientific evidence. |
| Related theme no.  1, 2, 3, 6, 7, 9, 10, 12, 14 | AIM:  To evaluate online self-learning modules on  critical appraisal skills to promote the use of research  in clinical practice among nurses |
| Jahromi B, Mosalanejad L. 2015. | Quasi-experimental study | None noted | 43 undergraduates taking a psychiatric course at Jahrom University of Medical Sciences | Review of various articles established that the concept of Web Quest  is an unknown one, not only in general education, but also in academic training and few studies have been conducted on this training tool and its consequences. | - Simple sampling was used to select the cases to be studied; the students entered the study through census and were trained according to Web Quest methodology; - Each topic included discussing concepts and then patient's treatment and the communicative principles for two weeks. - Active participation of the students in response to the scenario and introduced problem was equal to preparing scientific videos about the disease and collecting the latest medical treatment for the disease from the Internet. - Three questionnaires, including the self-directed learning Questionnaire, teamwork evaluation Questionnaire (value of team), and Buffard self-regulated Questionnaire, were the data gathering tools. | In view of Web Quest's positive impacts on students’ learning behaviors, problem solving and teamwork, the effective use of active learning and teaching practices and use of technology in medical education are recommended. |
| Related theme no.  1, 3, 4, 7, 9, 11, 12, 13 | AIM:  The aim of this study is to evaluate the Web Quest influence on students' learning behaviors. |
| Ramaswamy R, Leipzig R, Howe C, Sauvigne K, Usiak C, Soriano P.  2015. | Information sharing | The sponsors did not play a role in the design, methods, data collections, analysis, or preparation of paper. | No sample required as this article is about informing the reader on POGOe | No comparisons made | - Overview given on medical education in general | POGOe is home to more than 950 geriatrics educational materials that clinicians at most U.S. medical schools and many others around the world have created and use  POGOe differs from a conventional repository of educational materials by encouraging the repurposing of its content components to create new resources (e.g., web-GEMs, trigger tapes, quizzes) and to encourage educators to align their educational methodology better with their learners’ needs by mixing and matching content and curricula.  It provides clinician–educators with resources to address current concerns of the Liaison Committee on Medical Education and academic medical centers: competency-based education, decreasing lecture hours and increasing interactive learning, and finding time to prepare materials and teach in the midst of mandates to increase clinical productivity. |
| Related theme no.  3, 4, 6, 8 | AIM:  Informs on the POGOe resources a  robust compendium of instructional and assessment materials which allows educators to concentrate more on improving learner performance in practice and not simply on knowledge acquisition |
| Phillips B, Turnbull B, He F.  2017. | Prospective Study | Ethics approval for the study was obtained from the Charles Darwin  University Human Research Ethics Committee.  Permission to  utilize the SDLRSNE survey tool and use it in an electronic format was obtained. | The survey questionnaire was distributed to students via email and was sent from Qualtrics™ an on-line survey platform.  Participants clicked on an anonymous link to complete the survey.  A total of 750 invitations were distributed using Qualtrics™  489 responses were returned  82 responses completed only the demographic data and did not complete the SDLRSNE questionnaire.  407 responses were used in the analysis; a response rate of 54%. | Mixed evidence from studies of SDLR with nursing students,  a resurgent interest has arisen with changing learning and teaching approaches driven by factors such as technological development and on-line learning. | - An online survey questionnaire was utilised based on the Self-directed Learning Readiness Scale for Nursing Education. - In contrast to earlier work, the participant profile in this study was predominantly non-traditional and captured participants from all three years of the nursing programme | Results found no significant age or gender differences.  First year students demonstrated lower levels of self-directed learning readiness.  Unexpected results were demonstrated in the survey subscales in relation to previous qualifications.  Participants who already held post-graduate qualifications showed lower scores for Self-Management than those who held diploma qualifications,  Students who already held a bachelor's degree had the highest scores in Desire for Learning.  The study findings suggest that universities should not assume that SDL capability is dependent on mature age or length of exposure to tertiary study |
| Related theme no.  1, 9 | AIM:  To elicit the level of self-directed learning readiness (SDLR) among undergraduate nursing students and to elicit what differences existed in the levels of SDLR in relation to age, gender, academic year, and previous qualifications. |
| Reviriego E, Cidoncha M, Asua J, Gagnon M, Mateos M, Garate L, Gonzalez R, Lorenzo E.  2014. | Prospective study | Nurses participated in the study voluntarily.  It was understood that they had agreed to participate once they had been told about the objectives of the study and what participation would involve, given written approval and completed the pre-course questionnaire. | A sample of 50 nurses and 3 tutors was recruited.  Of the 50 health professionals recruited, 3 did not complete the course for personal or work-related reasons. | A literature search was conducted on educational interventions for critical appraisal skills.  The research team evaluated the contents and usefulness of Info Critique with a view to adapting it to their setting. | - Educational strategies and assessment instruments were established for the course. - A course website was created that contained contact details of the teaching team and a course handbook and videos introducing the course - Assessment used questionnaires before and after the course, in order to explore the main intervention outcomes: knowledge acquired and self-learning readiness. Satisfaction was also measured at the end of the course. - In general, participants’ performance on the knowledge questionnaire improved after the course. | Participants significantly improved their knowledge score and self-directed learning readiness after the educational intervention, and they were overall satisfied with the course.  For the health system and nursing professionals, this type of course has the potential to provide methodological tools for research, promote a research culture, and encourage critical thinking for evidence-based decision making.  Further, analyses confirmed statistically significant differences between pre- and post-course results (p < 0.001). With regard to self-learning readiness, after the course, participants reported a greater readiness and ability for self-directed learning. Lastly, in terms of level of satisfaction with the course, the mean score was 7 out of 10. |
| Related theme no.  1, 3, 4, 5, 6, 7, 9, 10, 11, 12, 13, 14 | AIM:  Was to train nurses from public Basqure Health service in critical appraisal, promoting continuous training and the use of research in clinical practice |
| Surr C and Gates C. 2017. | A critical synthesis including qualitative, quantitative  and mixed/multi- methods studies. | Not included | A total of 20 papers were included in the review, the majority of which were low or medium quality,  Impacting on general visibility. | The 16 different training programs evaluated in the studies varied in terms of duration and mode of delivery, most employed face-to-face didactic techniques.  Studies predominantly  reported on reactions to training and knowledge, only one study evaluated outcomes across all of the levels of the Kirkpatrick model. | - The review considered a range of knowledge, skills, competencies and qualities required of healthcare professionals and the need to prepare health professionals to be accomplished and responsible practitioners. - Kirkpatrick’s (1984, 1979) four level ‘Return on Investment’ model. Was applied: - Level 1: The learners’ reaction to and satisfaction the program - Level 2: The extent of learning including knowledge, skills, confidence and attitudes; - Level 3: The extent to which completion of the training leads to staff behaviour or practice change; - Level 4: The results or outcomes of training, in terms of quality of patient care. | This review has identified that previous systematic and literature reviews have failed to consider the impact of training design, content and delivery in considering benefits, instead using the term training to reflect a huge range of provision using varying designs, content and delivery methods, which are not directly comparable with one another.    This review has demonstrated that positive and negative features of training programme that may be more likely to lead to pedagogical, clinical or practice efficacy can be identified and suggestions made for the design of future training programmes for hospital staff. |
| Related theme no.  1,2,3,4,5,6,7, 9, 10, 11, 12, 13, 14 | AIM:  The purpose of this literature review was to examine published evidence on the most effective approaches to dementia training and education for hospital staff. |
| Surr C and Gates C.  2017. | Systematic review | Not included | In total 152 papers were included in the review | Comparison of four themes, drawn from 34 of the studies, of which the majority were moderate  or high quality emerged from our CIS:  1. Relevance of training to learners’ role and practice  2. Teaching and learning approaches adopted  3. The quality of the training materials  4. Trainer/educator qualities | - Data analysis was conducted using critical interpretive synthesis (CIS; Dixon-Woods et al., 2006), a nontraditional systematic review method that draws on systematic qualitative enquiry, incorporating interpretive approaches. - A further review of the studies reported on the impact of education and training on outcomes or results, for people with dementia, staff, and family caregivers/ relatives. - Kirkpatrick’s (1984, 1979) four level ‘Return on Investment’ model. Was applied   • Level 1: The learners’ reaction to and satisfaction the program  • Level 2: The extent of learning including knowledge, skills, confidence and attitudes;  • Level 3: The extent to which completion of the training leads to staff behaviour or practice change;  • Level 4: The results or outcomes of training, in terms of quality of patient care | Identified a number of key features in effective dementia training and which support understanding of approaches to effective professional development and workplace education more broadly.  Training/education most likely to be effective if:  • Relevant and realistic to the role, experience, and practice of learners  • Includes active participation  • Underpins practice-based learning with theoretical or knowledge-based content  • Ensures experiential and simulation-based learning includes adequate time for debriefing and discussion  • Is delivered by an experienced trainer/facilitator  • Does not involve reading written materials (paper or Web-based) or in-service learning as the sole teaching method  • Is of a total duration of 8+ hours with individual training sessions of at least 90 minutes  • Includes active, small, or large group face-to-face learning either alone or in addition to another learning approach  • Includes learning activities that support the application of training into practice  • Provides staff with a structured tool, method or practice guideline to underpin care practice |
| Related theme no.  1,2,3,4,5,6,7, 9, 10, 11, 12, 13, 14 | AIM:  The aims of this review were to identify the factors associated with effective dementia education and training for health and social care staff, across service settings. |
| Scerri A, Innes A, Scerri C.  2017. | Systematic review | None noted | Literature from five databases were searched, based on a number of inclusion criteria.  Initially 496 articles 53 duplicates removed 28 potential articles identified in the end 14 studies were selected  The selected studies were summarised and data was extracted and compared using narrative synthesis based on a set of pre-defined categories.  Methodological quality was assessed |  | - Fourteen peer-reviewed studies were identified with the majority being pre-test post-test investigations. - No randomised controlled trials were found. - Methodological quality was variable with selection bias being the major limitation. - There was a great variability in the development and mode of delivery although, interdisciplinary ward based, tailor-made, short sessions using experiential and active learning were the most utilised. - The majority of the studies mainly evaluated learning, with few studies evaluating changes in staff behaviour/practices and patients’ outcomes | High quality studies are needed that especially evaluate staff behaviours and patient outcomes and their sustainability over time. It also highlights measures that could be used to develop and deliver training programmes in hospital settings.  Hospital staff need to be better trained to improve their knowledge, confidence and attitudes in order to change behaviours and practice that can lead to better patient outcomes.  Various theoretical models and strategies can be used in the development of the training programme, although staff satisfaction to the programme is better where experiential, reflective and active learning is used and a training needs analysis is carried out.  Various methods of delivery can be used. Some benefits have been reported such as improved knowledge, confidence and better staff attitudes, the effectiveness of these training programmes is limited in relation to changes in staff behaviour and patient outcomes.  The need for further high-quality studies with extended follow-ups.  Training programmes need to be tailor-made to hospital settings as they may be different to residential homes |
| Related theme no.  1, 2, 3, 4, 5, 6, 7, 8, 9, 10, 11, 12, 13, 14 | AIM:  What is the current evidence on dementia training programmes directed to staff working in general hospital settings?  Specifically  1. What is the quality rating of the selected studies  2.What are the characteristics of the training programmes selected Based on  Kirkpatrick’s Evaluation Framework,  3.Effectiveness of these training programmes in developing and evaluating dementia training programmes |
| Lewis K, Cidon M, Seto T, Chen H, Mahan J.  2014. | Systematic review | Not included | 72 articles from 712 in original search' | Instructional effectiveness, improvement and satisfaction.  Outlined a comprehensive overview of study purposes and eLearning development linked to learning outcomes and disciplines | - Based on the study outcome measure, the results showed seven categories of study focus:   (1) instructional improvement,  (2) Instructional effectiveness,  (3) instructional effectiveness and satisfaction,  (4) instructional module development,  (5) instructional comparison and satisfaction,  (6) instructional feasibility, and  (7) Needs assessment. | 1. e-Learning activities need to be based on well- defined goals and objectives and driven by an appropriate needs’ assessment of the learners.  2. Situational factors regarding e-learning activities must be carefully considered: learner characteristics, technological resources and other important infrastructure, sustainability, and fit with other educational activities.  3. Methods of assessing learning and effectiveness of the instructional design must be formulated before e-learning modules/ activities are created.  4. e-Learning should incorporate methods known to promote critical thinking skills, reflective practices, and lifelong learning through frequent repetition, reinforcement, active engagement, and visualization in learning.  5. It should also employ cutting-edge technologies and interactive multimedia to accommodate different learning needs and individual learning preferences. |
| Related theme no.  1, 4, 5, 9, 13 & Kirkpatrick’s model | AIM:  Overview of the present state of E-Learning in medicine and how best to leverage educational value & effectiveness |
| Seckman C.  2014. | Descriptive correlational design | Permission granted by institutional review board and participants privacy & confidentiality secured | 96 nursing students volunteered to participate in the study | What is the relationship among perceived sense of community, cognitive engagement and learner outcomes for students enrolled in an internet-based course  Is there a difference between spring /fall learning outcomes?  What teaching /learning activities promote sense of community? | - Outcomes were measured using the final examinations scores and course grades as well as self- assessment; specific teaching/ learning activities that promote community. A survey called student characteristics and perceived learning outcomes was used. - Roavi’s classroom community scale was used | Promoting a sense of community & cognitive engagement has the potential to improve learning outcomes |
| Related theme no.  1, 3, 5, 6, 12, 13 | AIM:  Evaluate the relationship among a perceived sense of community, cognitive engagement and learner outcomes among undergraduate nurses enrolled in an internet-based learning course |
| Arving C, Wadensten B , Johansson B. 2014. | Descriptive study using open-ended questions and focus group interviews | Under Swedish law ethics approval not required | 53 RNs participated in focus group interview  32 responded to open ended questions. | Analysis of focus group interviews and response to 5 open-ended questions based on the opinions of a combination of lectures on campus vs online. | - Web-based questionnaire and focus group interviews which were recorded verbatim | Overall RNs pleased with web lectures, wanted more focus on nursing knowledge & practice; blended learning was appreciated including face to face they concluded that blended learning could be successful if content was more geared to advance nursing |
| Related theme no.  1, 3, 4, 9, 11, 12, 13 | AIM:  To describe RN's thoughts on blended learning format using open-ended questions and focus group interview.  Aim was to reduce education days on campus from 8 to 5 thus transferring lectures to an online format |
| Mgutshini T. 2013. | Qualitative comparative cross- sectional study | The University Institutional Review board approved the study and  Questionnaires were anonymous | 61 nursing students undertook 14-week course:  53 completed the two questionnaires:  23 in the campus-based course  30 in the online course | Real time head to head comparison of online learning and campus-based learning; Online learners reported having equal opportunity to express viewpoints and to be heard more than in the classroom setting | - A nine-item data recording form; a standardised student satisfaction questionnaire (SIR -II) was completed including items of satisfaction with tutor, peers, communications, discussion, access to support and convenience of learning options; both questionnaires were administered using Qualtrics | Online learning offered comparable content mastery which contradicts the general view that online teaching approaches are at best a complement to traditional face to face delivery; this article raises questions about long assumed superiority of campus-based learning over online study. |
| Related theme no.  1,5,8, 9,10, 11, 13 | AIM:  To present a synchronous head to head comparison between online and campus -based student experiences of an undergraduate course |
| Wearne S, Greenhill J, Berryman C, Sweet L.  2011. | Qualitative research using thematic analysis | Ethics obtained from Flinders University Behavioural & Social Science Committee | Australian study small sample  20 clinicians: GP's, Nurses, Pharmacist, paramedics, from rural/remote/metro location; | Comparison of convenience, engaging and affirming, meaningful learning amongst discipline; hit & run learning: internet challenges in remote settings, and IT literacy, juggle competing interest; workplace support; family support. | - Semi-structured in-depth interviews with 20 clinicians. Interviews transcribed verbatim into N-Vivo qualitive analysis software. - Data analysed against a template derived from open -ended coding merged with a priori themes from a program logic model | Results confirm the internet’s potential to develop clinical educators but also sounds caution that access is not automatic and needs active facilitation & multifaceted support; trade off individual convenient study and learning community; importance of discussion boards; importance of interaction and learning relationships; need for dialogue and feedback; protected study time is ideal but not always possible; |
| Related theme no.  3, 5, 6, 7, 11, 14 | AIM:  Understand how clinicians experience of online study affected their learning, the barriers they face and what supports assisted them |
| Moule P, Ward, R, Lockyer L.  2010. | Mixed methods approach primarily qualitative with a using a quantitative component | University of West England Bristol ethics committee gave approval addressing the issues of anonymity and confidentiality | Twenty-five Higher Education Institutions were visited  41 students took part in focus groups and  35 staff interviewed | Comparison of ‘Pedagogic use’ ‘Factors inhibiting use’ and ‘Facilitating factors in engagement’ where analysed by way of focus groups and questionnaires | - Questionnaire responses were coded and the data entered into SPSS and a transcript of the focus groups were analysed and developed key themes related to the student use and experience of e-learning | Students experienced a range of e-learning applications but mainly as a support to existing face to face delivery, suggesting that e-learning is used as an adjunct to existing modes of delivery and learning experiences |
| Related theme no.  3, 4, 5, 11, 13 | AIM:  Research on nursing and health care students’ experiences and use of e-Leaning |
| Berger J, Topp R, Davis L, Jones J, Stewart L.  2009. | Record review | Record review obtained from the Institutional Review board and the Norton Healthcare research office | Examination of education records of 1661 registered nurse from four hospitals | Comparison of face to face instructor led training program (ILT), an instructor -facilitated web-based training program (FWBT) or an independent web-based training program (WBT) | - Post-tests proficiency results, course evaluation results and costs. CHI-square statistics were employed for group comparisons involving categorical data and one-way analysis of variance statistics were calculated | 1544 chose (WBT) 67 (FWBT) 50 (ILT): 93% selected WBT indicating a preference for WBT most reported satisfaction for all 3 methods of instruction; equal effectiveness for competency the WBT was most cost effective due to the large numbers $4.05 pp compared to $110.20 ILT $59.35 FWBT; learning needs to be engaging & interactive |
| Related theme no.  1,3,4,5,8,9,11 | AIM:  To compare 3 different mechanisms of staff instruction concerning patient education in the inpatient setting |
| Ellis C, Rolann D, Blair M.  2013. | Literature review | Literature review | Only 4 papers met criteria; none of the 4 evaluated the translation of learning into practice;  A pre /post-test method used to assess level 1 & 2 | A comparison using Kirkpatrick’s evaluation model including: Reaction, Learning, Behaviour & Results: | - A review of the literature identified educational interventions designed to measure improvements in knowledge, attitudes & beliefs which can be relayed to improve immunisation uptake. - Only 4 papers met criteria; none of the 4 evaluated the translation of learning into practice; - A pre /post-test method used to assess level 1 & 2 | Following a review of the literature the following recommendations were made: evaluation must be built into the development stage to measure efficacy against objectives; evaluation should be independent of development; a perceived lack of expertise in evaluation or audit and complexities may curtail evaluation however most local sites have access to research institutes |
| Related theme no.  Kirkpatrick’s, 9, 12 | AIM:  Does the Healthy Child e-Learning (HCP) program improves practitioner knowledge and whether the increased knowledge has a positive measurable impact on clinical practice |
| Sowman A and Jenkins L.  2013. | Mixed Methodology | Approval obtained from Research Review board at the Hashemite University. | An intervention group of 25 in distance class  A control group 35 student in a hybrid class | Comparison between a distance learning experience and a hybrid class delivered as a 2 hr weekly face to face interactive lectures.  The same assignments, design questions and activities were used as in the distance class. | - SPSS version 16.0 was used to analyse results of an open-ended satisfaction questionnaire, based on ‘The Framework and Benchmark for Best Practice in Web-based Nursing Courses’ including: satisfaction of the distance learning, demographic data and course achievements, instructor support. | High satisfaction in the distance course, main advantage was sense of control, enjoyment, improvements in learning, communication & computer skills; The mean final grade of the distance group was higher than the hybrid group. Distance students were overwhelmingly satisfied with the course and achieved better than their hybrid class; most pleased about continuous support, prompt feedback and contact from instructor |
| Related theme no.  3, 4, 6, 13 | AIM:  Describe the design, delivery processes of distance education within these 3 principles:  1. need for course accessibility & appropriate navigation  2. communication & interaction among students with instructor  3. emphasis on active & collaborative learning |
| Carroll C, Booth A, Papaioannou D, Sutton A, Wong R**.** 2009. | Systematic review | none | 107 potentially relevant full papers were retrieved and examined to determine whether they satisfied the inclusion criteria. | A systematic review of qualitative data reporting UK health professionals’ experiences of the ways in which on-line learning is delivered.  Evidence synthesis was performed with the use of thematic analysis grounded in the data | - A search strategy was constructed to identify relevant studies with the use of free text and, where available, database thesaurus terms, representing e-learning ~such as “online learning,” “virtual learning”! and all work sectors such as “staff” or “profession”! in order to capture any potential study that may include health professionals. - The team drew up a refined and mutually agreed framework of themes. - The aim of this stage was to generate a new thematic framework to describe and explain health professionals’ experience of e-learning. | If on-line courses seek to enhance health professionals’ experience of e-learning, and consequently improve the effectiveness of on-line delivery, course providers need to take careful account of presentation and course design.  They must also provide flexibility, offer means for both support and rapid assessment, and develop effective and efficient means  Five key themes emerged from the data: peer communication, flexibility, support, knowledge validation, and course presentation and design. |
| Related theme no.  2, 3, 4, 5, 7, 9, 11, 12, 13 | AIM:  Which e-learning techniques most enhance the  learning experience of health professionals in the United Kingdom |
| Kontio R, Hatonen H, Joffe G, Pitkanen A, Lahti M, Valimaki M. 2013. | A randomized controlled  study, | Ethics committee various organisations involved | 137 completers (those who participated in the 9-month follow-up assessment). | 12 wards were randomly assigned to the ePsychNurse.Net (intervention) or training as usual (control).  Baseline and 9-month follow-up data on nurses’ knowledge  of coercion-related legislation, physical restraint and seclusion, their attitudes towards  physical restraint and seclusion, job satisfaction and general self-efficacy were analysed | - The measures used were the Knowledge of Legislation (Immonen 2005), Physical Restraint Questionnaire/Knowledge Scale (Janelli *et al*. 1992), - Seclusion Questionnaire/Knowledge Scale and its modified version (2007). - The measures used were, respectively, Job Satisfaction Scale (JDS) (Hackman & Oldman (1974) and modified by Vartiainen (1986), and General Self-Efficacy Scale (Jerusalem & Schwarzer 1992). | No between-group differences were found on any variable, with the exception of a change in attitude to seclusion in favour of the control group.  The findings of the long-term effects did not differ from the immediate outcomes (3-month follow-up) and the improved level of knowledge acquired and further consolidation of that knowledge did not take place in the 6-month period after the 3-month ePsychNurse.Net course. |
| Related theme no.  8,13,14 | AIM:  To explore the long-term impact of an eLearning course on psychiatric nurses’ professional competence in practicing seclusion and restraint and on their job satisfaction and general self-efficacy at 9-month follow-up. |
| Sherman H, Comer L, Putnam L, Freeman H. 2012. | Randomised control trial | Approved by hospital and university review boards | 68 subjects | Demographics were compiled and compared using Fisher’s exact test or pooled t test. Pretest and posttest results were analyzed for central tendencies and were compared between groups using paired t-test analysis. Finally, pooled t tests compared demographics with posttest scores for analysis. | - The study used instruments to measure demographics, cognitive learning, and education effectiveness. - The 46-item posttest was used. - The instrument consisted of multiple choice, true/false, short essay, and calculation questions. | This study provided experimentally derived evidence regarding effectiveness of blended versus traditional lecture for critical care pharmacology education. Regardless of learner demographics, the findings determined no significant differences in cognitive learning outcomes or learner satisfaction between blended versus lecture formats. |
| Related theme no.  1,3,5,6,7,11 | AIM:  To identify learning outcomes and student satisfaction associated with blended versus traditional lecture classroom learning of critical care pharmacology nursing continuing education. |
| Du S, Liu Z, Liu S, Yin H, Xu G, Zhang H, Wang, A.  2013. | A systematic review | Not addressed | 69 articles were reviewed  9 studies were considered eligible based on PICO Population, Intervention, Comparison, Outcome | A comparison of the 9 studies considered six items: randomization, allocation, concealment, blinding, dropout/attrition, intention to treat analysis and baseline comparability | - Selected studies were scored using a quality critical appraisal list for RCTs recommended by the Cochrane Handbook for Systematic Reviews of Interventions | Main outcome measures included knowledge, skill performance participant’s satisfaction and self- efficacy;  The study showed a positive role of web-based learning, compared with controls; web-based education has equivalent or even better effects in improving participant’s knowledge & skills performance & improving self- efficacy in performing nursing skills. Participants expressed high satisfaction toward online learning |
| Related theme no.  1,3,4,5,6,12,13,14 | AIM:  Examine the efficacy of web-based distance education for nursing students in terms of knowledge and skills and to explore the effectiveness of web-based distance learning. |
| Innes A, Kelly F, McCabe L.  2012. | Cross-sectional survey | Study approved by the School of applied Social Science Board of Ethics Bournemouth UK | 76 students completed the survey (30%) of the 250 enrolled in dementia studies program | Comparison of students’ views on learning with a range of methods: face to face, web-based, reflection and telephone or video conferencing vs 14 weeks online delivery and learning into practice. | - Online survey using Survey Monkey | Students in the dementia field value flexibility offered by blended learning; online learning offers a way to study and work at the same time.  Students adopted a reflective approach to their learning & practice and have applied new knowledge to their practice; making connections of learning to practice, passing information to others, the study reported on the ability to create leaders who could join the abilities needed to care and abilities to manage who ultimately motivates the workforce to deliver the hands on care to people with dementia |
| Related theme no.  1, 5, 6, 8, 9, 13, 14 | AIM:  Evaluation via an online questionnaire, of student views of the delivery modes and learning impact for the first online postgraduate program in dementia studies worldwide. |
| Surr C, Smith S, Crossland B, Robins J.  2015. | A repeated measures design | Ethics granted by the humanities, social and Health Sciences Research Panel University of Bradford | 41 Acute hospital staff working in clinical roles the majority of whom were nurses | Compare training effect on staff attitude and satisfaction of working with people with dementia and their beliefs about their own caring efficacy | - Analysis conducted using SPSS -PC software. - Repeated measure design to capture impact of training over a period of time - Measures at baseline repeated 4-6 weeks and then 3-4 months 3 measures used:   - The Approach to Dementia Questionnaire (ADQ);   - The Staff Experience of Working with Dementia Residents Scale;   - The Caring Efficacy Scale. 41 staff completed | The training program was effective in producing a significant positive change on all three outcome measures following intermediate training compared to baseline.  Producing significant improvements in attitudes towards and satisfaction in caring for people with dementia and feelings of caring efficacy. |
| Related theme no.  9, 12 | AIM:  Evaluate the efficacy of a specialised training program for acute hospital staff regarding improving attitudes, satisfaction and feelings of caring efficacy, in provision of care to people with dementia |
| Chao S, Chang Y, Yang S, Clark M. 2017. | Quasi-experimental design | Approved by the Kuan Tien hospital IRB Committee | 100 students  51 in experimental group &  49 in control | The project occurred in 2 phases:  1^st^ phases involved creating an interactive situational e-learning system and integrating it into the ethics course;  2^nd^ phase implemented the modified course and studied its effects on students ethical decision-making abilities | - Pre-test / post-test questionnaires used, forum discussions, Descriptive and inferential statistical analysis using SPSS22.0 - Generalised Estimating Equations method | The interactive situational e-learning system developed by our project was helpful in developing the students’ competence in ethical reasoning.  The e-learning system and the situational teaching materials used in this study maybe applicable in nursing and related professional ethics courses |
| Related theme no.  1, 3, 12 | AIM:  To develop & implement an interactive situational e-learning system, integrating nursing ethical decisions into a nursing ethics course and to evaluate the effects on student nurse’s ethical decision- making competence. |
| Cartwright J, Franklin D, Forman D, Freegard H.  2015. | A mixed methods research design used | The Curtin University Human  Research Ethics Committee approved the research. | A total of 125 students from five health sciences disciplines at  Curtin University participated in the online IPE dementia  case study | Comparison of the pre- and  post-mean differences for each of the three sub-scales of the  ISVS; self-perceived ability to work with others, value in working with others and comfort in working with others. | - An Adapted version of Interprofessional Socialization and Valuing Scale *(ISVS)* completed pre and post the online case study and via thematic analysis of free text responses | The online IPE case study was successful in developing the collaborative mindsets and interprofessional capabilities required by a future workforce to meet the complex, client-centred needs of people living with dementia. |
| Related theme no.  1, 3, 6 | AIM:  To develop, implement and evaluate an online interprofessional education (IPE) dementia case study for health science students. care. |
| López Soblechero M, González Gaya C, Hernández Ramírez J.  2014. | A comparative study |  |  |  |  |  |
| Related theme no.  4, 5, 6, 11, 12, 13 | AIM:  1. Evaluate the distance model of official vocational education and training offered by means of a virtual learning platform.  2. establish that both on-site classroom and online distance modes of vocational education and training can be seen as complementary | The Alfonso de Avellaneda Vocational Education and Training School, faculty board | Data and results gathered over the course of eleven academic years for 1,133 of our students enrolled in an official vocational education and training program | We offered classes both in traditional classroom mode and through online distance learning. | - A series of surveys using the COLLES method and the ‘Quality on the Line’ model was used to evaluate the distance learning system. - A descriptive analysis of the variables studied; inferential statistical techniques are subsequently applied in order to study the relationships that help form the basis for the conclusions reached. | This study's results provide evidence that a broad offering of vocational education and training opportunities will facilitate access to such learning for students who require it, regardless of their age, employment status, or personal circumstances, with the online distance mode playing a fundamental role while also yielding results equivalent to those observed for classroom instruction. |
| Riley K and Schmidt D.  2015. | A qualitative study based on appreciative  inquiry methodology using semi-structured interviews  . | Ethics approval was received from the ethics committee of the local NSW health service. | Fourteen nurses were involved in the study, including Managers,  Clinical Nurse Specialists Registered Nurses, Enrolled Nurses and  Assistant in Nursing | A comparison of Discovery (what works), Dream (imagine what might be), Design (what should be) and Deliver (what will be). Participants were asked what they felt worked well | - This study used Appreciative Inquiry (AI), an action research methodology that aims to give a voice to positive factors by focusing on what is possible and identifying the reasons for success. - AI consists of four phases: Discovery (what works), Dream (imagine what might be), Design (what should be) and Deliver (what will be). - Interviews were digitally recorded and transcribed and coded manually by the principal researcher using a coding table that matched AI | This study demonstrates that rural nurses’ engagement with online learning would be enhanced by a whole of system redesign in order to deliver a learning environment that will increase satisfaction, engagement and learning outcomes.  Online learning needs to be designed not just in terms of modules and content, but an entire educational experience. |
| Related theme no.  2, 3, 6, 5,7, 8, 13, 14 | AIM:  To explore the factors that influence rural nurses’ engagement with online learning within a rural health district in (NSW) Australia |
| Booth A, Carroll C, Papaioannou D, Sutton A, Wong R. 2009. | A systematic review | Not addresses | A total of 3476 references were retrieved  Twenty-nine studies were included in the health subset of the review. | A comparison framework emerged including Applicability, Attractiveness, Usability, Offline working, Asynchronous engagement, Learner interaction, Peer support, Moderated learning, Formal support, Assessment | - Standard checklists of quality assessment criteria, for different types of study design, were used to appraise the quality of the included studies. - Where appropriate, case study and survey or questionnaire critical appraisal checklists were used to assess the quality of a specific study design in more detail. - The quantitative and qualitative checklists from the Alberta Heritage Foundation for Medical Research were used. | Five broad themes were identified from the 29 included studies:  (i) peer communication;  (ii) flexibility;  (iii) support;  (iv) knowledge validation; and  (v) course presentation and design.  These broad themes were supported by a total of eleven sub-themes. Components from the FOLIO Programme were analysed and existing and proposed developments were mapped against each subtheme.  This provides a valuable framework for ongoing course development. |
| Related theme no.  1, 5, 9, 11 | AIM:  A systematic review of the UK published literature on e-learning  in the health workplace and to apply the findings to one of the most prolific UK  e-learning initiatives in the health sector—the  (FOLIO) Programme |
| Page J, Meehan-Andrews T, Weerakkody N, Hughes D, Rathner J.  2017. | A comparative cross-sectional study | The study was approved by La Trobe University Human Ethics Committee | 437 responses | A Comparison of student’s perceptions of learning and support and tools; attitude toward pre-workshop quizzes; usage of learning resources; attitude toward blended learning; suggestions on improvements to subject; time spent on subject | - Statistical tests were performed in Microsoft Excel 2013, using the Analysis Tool Pak add-in. - Single Factor ANOVA compared student ATAR and marks for first-year physiology. As well as thematic analysis | The data suggest that there is a quantifiable benefit to didactic teaching in the blended teaching mode that is not reproduced in online self-directed learning, even when face-to-face guided inquiry-based learning is embedded in the subject. |
| Related theme no.  4, 5, 7, 9, 11 | AIM:  To assess the impact of online learning resources on the students’ perceptions of their learning experiences  To evaluate the impact of the changed pedagogy on the students’ learning outcomes  To identify teaching and learning resources utilized that were found to be most beneficial to and to track the durability of these resources over time. |
| Mayer B, Ring C, Muche R, Rothenbacher D, Schmidt‑Straßburger U.  2017. | An evaluation report on a web‑based online master  programme | Not addressed | There were 18 students in the initial cohort (Group A) and  23 students in the second and third cohorts combined (Group B); | A comparison of several teaching–learning modalities:  Audibly via didactic videos, visually via the presentation slides, in writing through the review articles and practically via exercises and multiple choice  questions | - Statistical tests: Principle of inferential statistical tests, Chi‑square test, t‑test, Wilcoxon test, McNemar test & Lecture evaluations based on specific questions concerning learning environment and information learned, each measured on a five‑point Likert scale. | We conclude that e‑learning of medical biometry is possible but is enhanced by supplementing on‑line modalities with some face‑to‑face interactions of lecturers and students in a blended learning approach. |
| Related theme no.  1,2,3, 4, 9, 11, 12, 13, 14 | AIM:  Describe the challenges in developing an e‑learning module using the example of a medical biometry course. |
| Ollerenshaw A, Wong Shee A, Yates M.  2018. | Quantitative pilot study using surveys and Google Analytics. | This study was approved by the Federation University  Australia Human Research and Ethics Committee  (Evaluation of the DPT; Project Number A14-  098). | Two hundred and sixty-three GPs and 160 practice nurses were invited to participate, with 42 respondents (GPs, n = 21; practice nurses, n = 21). | This pilot study  evaluated the accessibility and utility of the online  DPT for health practitioners in regional Victoria.  Health practitioners valued the content and the availability of local resources and reported improvements in knowledge, skills and confidence in dementia management. | - Descriptive analysis was used for the survey and Google Analytics data. Survey questions that elicited free text responses were reviewed and independently categorised by two researchers. - The survey contained 40 questions including open and closed questions, Likert scales, and multiple choice questions | Primary health practitioners reported that the dementia pathways tool provided access to region specific referral and management resources for all stages of dementia.  Such tools have broad transferability in other health areas with further research needed to determine their contribution to learning in the practice setting and over time. |
| Related theme no.  2, 6, 7, 9, 10, 12. 14 | AIM:  To explore the awareness and usage of an online dementia pathways tool for primary health practitioners (GPs and nurses) in regional Victoria. |
| Kirkpatrick J and Kirkpatrick W. 2009. | Training Program | none | Fifty year history of delivering this model of training | Recognised in much of the literature as a successful model of training | - Trainers must begin with desired results and then determine what behaviour is needed to accomplish them. - Then trainers must determine the attitudes, knowledge, and skills that are necessary to bring about the desired behaviours. - The final challenge is to present the training program in a way that enables the participants not only to learn what they need to know but also to react favourably to the program.” Kirkpatrick | Established 4 levels of successful Training  Level 4 RESULTS: To what degree targeted outcomes occur, as a result of the learning event(s) and subsequent reinforcement.  Level 3 BEHAVIOUR: To what degree participants apply what they learned during training when they are back on the job  Level 2 LEARNING: To what degree participants acquire the intended knowledge, skills, and attitudes based on their participation in the learning event  Level 1 REACTION: To what degree participants react favourably to the learning event |
| Related theme no.  1, 2, 7, 8, 9, 11, 12, 14 | AIM:  Description of the cornerstones of the business partnership model of training and the keys to successful implementation of training programs. |
| Collier E, Knifton C, Surr C.  2015. | Contemporary issues piece | none | The Higher Education for Dementia Network  brings together academics involved in dementia education to discuss issues  The network currently includes representatives from 53 Universities across the UK. | Issues piece | - The Higher Education Dementia Network curriculum provides a guideline for content and highlights the absence of a standardised curriculum in the UK. Standardised curricula may offer a pragmatic approach to ensuring national compliance, but it is not currently known what type of approach works best for dementia education and whether a single or multiple curriculum will be required for a highly diverse health and social care workforce | Currently there are no ways of standardising the expertise, knowledge and skills of dementia lecturers at a national level in the UK.  Ultimately many Education Institutions may find the pressure to ‘do it’ will take precedence over ‘doing it well’.  Doing it well will need the health and social care workforce to have the necessary knowledge, skills and competence so that those who live with the condition can be confident in their future care. |
| Related theme no.  1, 2, 5, 6, 10, 11, 14 | AIM:  This paper raises a question of by whom education for dementia workers is best  provided. |
| Kirkpatrick J and Kirkpatrick W. 1998. | Book | nil | 50-year application of the model | nil | - The four levels of Kirkpatrick's evaluation model measure:   · reaction of student  · learning  · behaviour  · results | Kirkpatrick’s theory has now become  arguably the most widely used and popular model for the evaluation of training and learning |
| Related theme no.  1, 2, 7, 8, 9, 12, 14 | AIM:  The four levels of Kirkpatrick’s training evaluation model |
