# Supplementary material for: A Web-Based Dementia Education Program and its Application to an Australian Web-Based Dementia Care Competency and Training Network: Integrative Systematic Review
Source: J Med Internet Res. 2020 Jan 22;22(1):e16808. doi: 10.2196/16808 (PMC7003124; doi:10.2196/16808)
Supplement: Supplementary file 2 [file jmir_v22i1e16808_app1.doc]

Example Ovid, Medline database Search Strategy to identify papers for review		Step	Search term		1.	Computer‐Assisted Instruction/ ﴾9060﴿		2.	Education, Distance/ ﴾3321﴿		3.	exp Education, Continuing/ ﴾35687﴿		4.	Learning/ ﴾38224)		5.	3 or 4 ﴾72908﴿		6.	Online Systems/ ﴾5822﴿		7.	computer communication networks/ or internet/ or local area networks/ ﴾73466﴿		8.	6 or 7 ﴾77945﴿		9.	5 and 8 ﴾2853﴿		10.	1 or 2 or 9 ﴾13253﴿		11.	limit 10 to English language ﴾12498﴿		12.	﴾self adj directed﴿.mp. [mp=title, abstract, original title, name of substance word, subject heading word,		13.	"outcome and process assessment ﴾health care﴿"/ or "outcome assessment ﴾health care﴿"/ or "process assessment ﴾health care﴿"/ ﴾81368﴿		14.	12 or 13 ﴾160200﴿		15.	11 and 14 ﴾1788﴿		16.	exp Dementia/ ﴾114458﴿		17.	15 and 16 ﴾9﴿		18.	15 not 17 ﴾1779﴿		19.	exp nursing/ or nursing, practical/ ﴾136080﴿		20.	exp nurses/ or exp nursing staff/ ﴾88816﴿		21.	19 or 20 ﴾195940﴿		22.	18 and 21 ﴾479﴿		23.	limit 22 to yr="2015 ‐Current" ﴾17﴿		24.	22 not 23 ﴾462﴿		25.	limit 24 to yr="2012 ‐ 2014" ﴾28﴿		26.	24 not 25 ﴾434﴿		27.	limit 26 to yr="2009 ‐ 2011" ﴾98﴿		28.	26 not 27 ﴾336﴿		29.	Plus a further 206 identified from alternative sources (542)
